# Position-Dependent Segmental Relaxation in Bottlebrush Polymers

**DOI:** 10.1021/acs.macromol.4c00243

**Published:** 2024-05-11

**Authors:** Karin J. Bichler, Bruno Jakobi, Alice Klapproth, Richard A. Mole, Gerald J. Schneider

**Affiliations:** † Department of Chemistry, Louisiana State University, Baton Rouge, Louisiana 70803, United States; ‡ Australian Nuclear Science and Technology Organisation, New Illawarra Road, Lucas Heights 2234, NSW, Australia; § Department of Physics & Astronomy, Louisiana State University, Baton Rouge, Louisiana 70803, United States

## Abstract

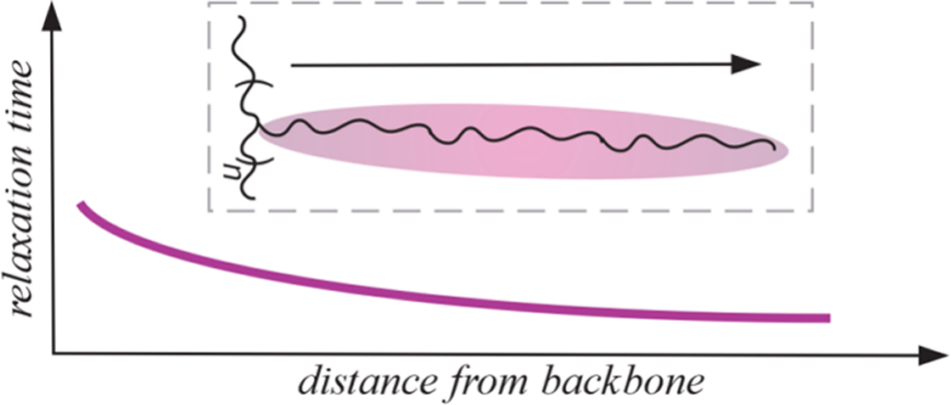

Segmental dynamics of specifically labeled poly(propylene oxide), PPO, based bottlebrush polymers, PNB-*g*-PPO, were studied using quasi-elastic neutron scattering. The focus was set to different parts of the side chains to investigate the hypothetical gradual relaxation behavior within the side chains of a bottlebrush polymer. Different sections of the side chains were highlighted for QENS via sequential polymerization of protonated and deuterated monomers to allow the study of the relaxation behavior of the inner and outer parts of the side chain separately. A comparison of these two parts reveals a slowdown due to the grafting process happening across the different regions. This is seen for the segmental relaxation time as well as on the time-dependent mean-square displacement. Additionally, the non-Gaussian parameter, α, shows a decreasing difference from Gaussian behavior with the distance to the backbone. Altogether, this leads to the conclusion that gradual relaxation behavior exists.

## Introduction

Understanding the dynamic behavior of polymers is very important for a deeper knowledge of their materials’ characteristics and properties. Bottlebrush polymers show extraordinary viscoelastic behavior.^1−3^ Therefore, the dynamics of bottlebrush polymers became the subject of several studies.^4−9^ In bottlebrush polymers, linear side chains are covalently bonded to a linear backbone. High grafting density of side chains leads to chain stretching, resembling a brush-like structure. This morphology can be tuned into elongated as well as spherical objects by changing the size ratio of backbone to side chain.^5,10,11^ Tethering one end of a linear chain changes the dynamics, including the segmental motion.^5^ Previous studies on homo- and hetero-bottlebrush polymers showed a slower relaxation of tethered side chains than the untethered chains. This observation is dependent on the length of the side chain. The slowdown is strongest for short side chains and decreases with increasing length and eventually yield equal relaxation times.^5^ However, the backbone experiences a plasticizer effect with a faster motion.^5,12^

More details were revealed by quasi-elastic neutron scattering (QENS) experiments on fully protonated bottlebrush polymers that indicate a stronger length scale dependence for shorter side chains. This points to heterogeneous dynamics over the entire *Q* range of the experiments.^4^ Both studies suggest a side-chain length-dependent gradual relaxation behavior in which the segmental relaxation time of the side chains seems to change systematically with the distance from the backbone. The innermost segments are likely to experience the strongest influence from the grafting point, which would explain why the effect diminishes for sufficiently long side chains. This results in the hypothesis that gradual relaxation behavior exists within the side chains. In the presented study, we are focusing on this fact more in detail to test whether such a gradual relaxation behavior exists in bottlebrush polymers.

For this purpose, partially deuterated bottlebrush polymers were synthesized to highlight specific parts of the side chains, using the difference in the incoherent scattering cross sections of deuterium and hydrogen for labeling in quasi-elastic neutron scattering (QENS). Having labeled parts in the inner or outer sections of the side chains allows to draw conclusions about the dynamical behavior along the side chains and finally about the hypothesis of the gradual relaxation behavior. More specifically, we have used bottlebrush polymers with 10 protonated units in the side chains located either at the grafting point or at the dangling end, while the remaining 14 or 17 units are deuterated to minimize their scattering contribution for QENS (Table 1).

**Table 1:**
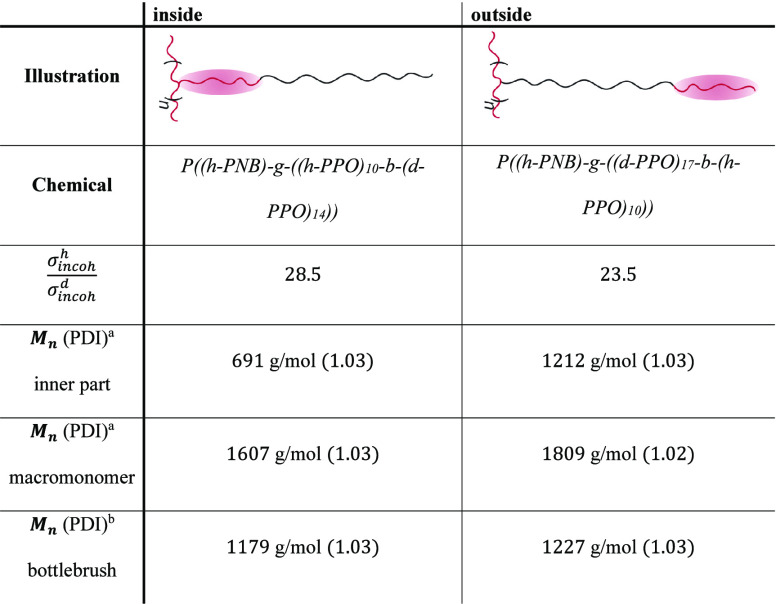
Schematic Illustration of the Two Different Samples Together with the Respective Sample Names, the Ratio of σ_incoh_^h^/σ_incoh_^d^, Their Number Averaged Molecular Weight, and Dispersity

a Determined by MALDI-TOF.

b Determined by GPC. Red color indicates protonated parts, whereas black color indicates deuterated parts.

## Theory and Analysis

Quasi-elastic neutron scattering measures the dynamic structure factor, *S*(*Q*,ω), depending on the time and length scale, *Q*, whereby the former part is encoded in the energy transfer, ω. In total, *S*(*Q*,ω) is a composition of coherent and incoherent parts, .^4,13−15^ Despite the contrast between the protonated and deuterated parts of our samples, the incoherent cross section of the protonated parts still dominates within the available *Q* range. This is particularly evident by comparing the incoherent scattering cross sections of the different parts of the side chains. Taking 10 protonated units ( cm^–1^) in relation to 14 or 17 deuterated units ( cm^–1^) results in the ratio  = 28.5 (Table 1). Therefore, the measured dynamic structure factor is , also known as the self-correlation function, giving information about the segmental dynamics and faster processes like methyl group dynamics, in the case of polymers.

The experiment provides , which is a convolution of  with the instrumental resolution, . This turns into multiplication if Fourier transformed into the time domain leading to the intermediate scattering function, . However, only  or  contains dynamical information on the sample.
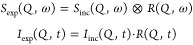
1For analysis in the time domain,  can be simply extracted by a division with the resolution function, *R*(*Q*,*t*)
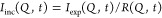
2whereas analysis in the frequency domain requires a numerical convolution of the model function with the instrumental resolution. For convenience, .

In this work, we focus on the analysis of the intermediate scattering function in the time domain, which allows the combination of different spectrometers to enlarge the available time range more easily. This has the advantage of covering dynamic processes over an extended time range, which is essential for polymers. As shown in the Analysis section, in the available time range, *t* ∼ 1 ps to *t* ∼ 1.5 ns, together with the selected temperatures, we cover segmental dynamics, similar as reported previously.^16^

The studies of partially deuterated side chains continue the previous QENS studies of fully protonated side chains. Therefore, a similar approach was used to analyze the experimental data. This includes a stretched exponential function for the segmental relaxation with relaxation time, , and stretching parameter, .

However, due to the partial deuteration, the ratio of protons in the side chains vs backbone is reduced compared to a previous study^16^ with 17% of total protons situated in the backbone. Their slower motions are outside the available time range and therefore can be treated as an elastic contribution. Accounting for that, we introduce the elastic contribution (EC), which is not participating in the dynamics.
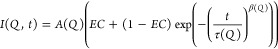
3As seen in eq 3, the relaxation time, , is associated with the stretching parameter, . For a better comparison across the two samples, we will use the average relaxation time, , calculated as
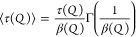
4which takes the contributions of  into account.^17^

Furthermore, the mean-square displacement will be evaluated by applying eq 5 to the intermediate scatting function in a  vs *Q*^2^ representation, leading to the mean-square displacement, , and the non-Gaussian parameter, , as fit parameters.
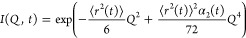
5

## Experimental Section

**Scheme 1:**
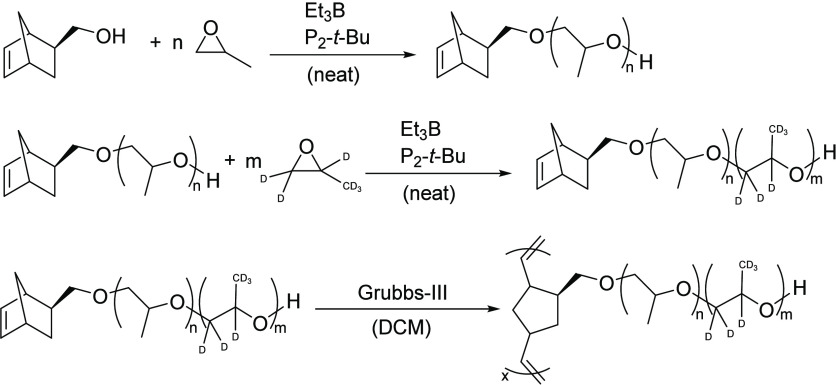
Synthetic Route toward Labeled PNB-*g*-PPO Macromonomers and Bottlebrushes on the Example of the Inside Labeled Bottlebrush

The macromonomers were synthesized following Jakobi et al.^18^ in two separate polymerization steps—first for the inner part initiated by exo-5-norbornene- 2-methanol and after purification and drying initiated by the hydroxy chain end (see Scheme 1).^18^ A direct block copolymerization was not possible due to precipitation of the catalyst/base after completion of the reaction due to the absence of solvent.

**Figure 1:**
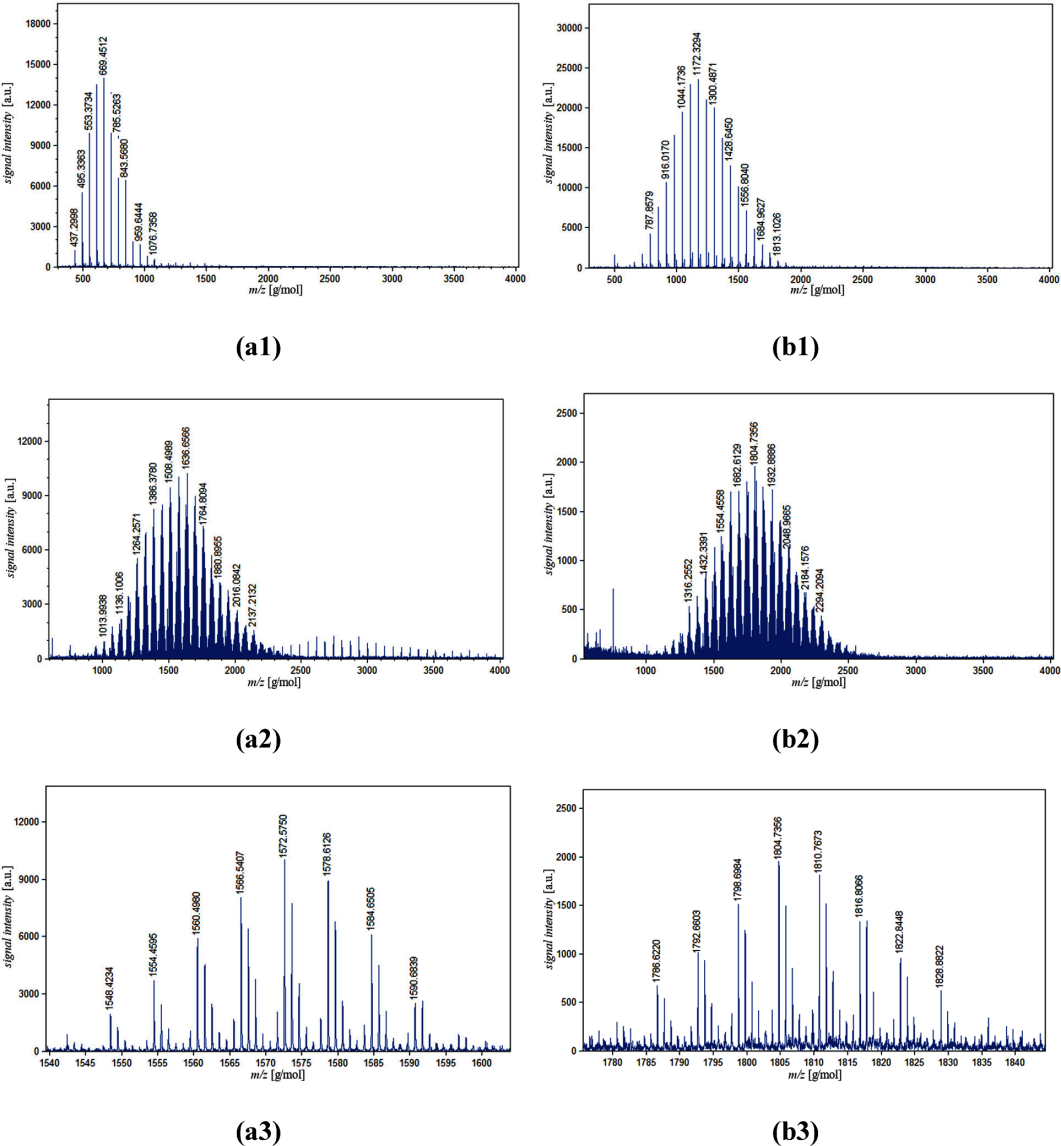
MALDI spectra of the inner part (top) of the labeled macromonomers, the complete macromonomers (middle), and zoom in (bottom) for the samples inside (a1, a2, a3) and outside (b1, b2, b3).

The blue traces in Figure 1 show the matrix-assisted laser desorption/ionization (MALDI) mass spectrometry of the inner part and the complete macromonomer of the samples with the protonated part of the side chains inside and outside. For the first step of the block copolymers, only one series of peaks can be identified, spaced either 58 g/mol, in the case of the inside label (a1), or 64 g/mol, outside label (a2), apart. In both cases the mass values correspond to an initiation with exo-5-norbornene- 2-methanol in combination with a sodium ion. After the second polymerization a more complex pattern is shown for both macromonomers.

The trace shows groupings of peaks spaced apart between 58 and 64 g/mol. Focusing only on one grouping of peaks (c1 and c2) shows a spacing of 6 g/mol, equivalent to the difference between the deuterated and protonated monomers. The spacing between groupings can be attributed to the difference between the combined number of repeating units in the macromonomers. The spacing inside a grouping resembles a constant combined number of repeating units with a different ratio of deuterated and protonated units. This assessment is confirmed by ^1^H NMR and the shifting singular peaks of the gel permeation chromatography (GPC) chromatogram (see the Supporting Information). The molecular weight characteristics as well as an illustration of the different parts highlighted for neutron scattering are listed in Table 1.

The glass transition temperature of protonated PNB is around *T*_g_∼ 310–320 K, which indicates that the dynamics of the PNB backbone is outside the frequency/time window of our QENS experiment.^19^ The relaxation time at *T*_g_ is roughly τ ∼100 s, whereas backscattering measures up to 3.8 ns, resulting in a relaxation time of PNB that is a factor of 100–10000 slower than the dynamics that can be measured with the instrument.^20,21^ Therefore, any relaxation contribution arising from the protonated PNB backbone itself is far outside the experimental time/frequency window, and thus, the relaxation signal is dominated by the side chains only. However, the protons located in the backbone lead to an elastic contribution, which was taken into account.

### Neutron Backscattering Instrument—Emu

Quasi-elastic neutron scattering experiments have been done at the neutron backscattering instrument Emu located at ANSTO, Lucas Heights, Australia.^22^ A wavelength of λ = 6.28 Å selected by a pyrolytic graphite crystal premonochromator via its (002) Bragg reflection was used. The instrument has a resolution of  μeV with a maximum energy range of *E* = −28 to 28 μeV or a corresponding Fourier time range of  ns. The *Q* range used was  Å^–1^ binned in increments of  Å^–1^. For shifting the segmental dynamics within the time window of QENS, temperatures of *T* = 325 K and *T* = 350 K were measured. The samples measured at *T* < 30 K were used as instrument resolution for data reduction. Additionally, measurements of the empty can as well as of the empty cryostat and vanadium for detector efficiency were also included in the data reduction. Therefore, no additional background was assumed during data analysis. The temperature was adjusted by a closed-cycle cryostat with a secondary helium circulation loop controlled by a needle valve. The samples were loaded in cylindrical annular aluminum cans with gap size of 0.2 mm, ensuring 90% transmission. Additionally, an aluminum heat shield was used to minimize the temperature gradient across the sample. For data reduction and analysis, the program MANTID was used.^23,24^

### Neutron Time-of-Flight Instrument—Pelican

Time-of-flight experiments were performed at the instrument Pelican, located at ANSTO, Lucas Heights, Australia.^25^ The setup using a wavelength of λ = 6 Å with an instrument resolution of  μeV was used, giving the theoretically accessible time range of 1–63 ps. The used *Q* range of *Q* = 0.2–1.8 Å^–1^ was binned in increments of  Å^–1^ to combine it with the backscattering experiment. The instrument resolution was measured at *T* < 30 K using the respective samples. To reach the measurement temperatures of *T* = 325 K and *T* = 350 K a closed-cycle cryostat with a secondary helium circulation loop controlled by a needle valve was used. The samples were loaded in cylindrical annular aluminum cans with gap size of 0.2 mm, ensuring 90% transmission. Additionally, an aluminum heat shield was used to minimize the temperature gradient across the sample. All the data reduction was done similar to Emu using MANTID.^23,24^

In order to combine the two different instruments, all data were Fourier transformed to the time domain by using MANTID.^23,24^ Subsequentially, the backscattering data are renormalized according to the time-of-flight data.

### Gel-Permeation Chromatography (GPC)

For all chromatography, an Agilent 1260 Infinity II GPC with a dRI detector, an isocratic pump, and an autosampler in combination with three MZ Analysentechnik columns, and a Wyatt Dawn Helios II detector, running with THF at 1 mL/min as the eluent, was used. The data were collected and analyzed with the Astra software package.

### Matrix-Assisted Laser Desorption/Ionization Mass Spectrometry (MALDI)

For all mass spectrometry measurements, a Bruker UltrafleXtrme MALDI TOF/TOF spectrometer was used. The samples were suspended in DCM at a concentration of 1 mg/mL and mixed with a DCTB solution (20 mg/mL in DCM) at a matrix:analyte ratio of 20:1. One microliter of the suspension was used for deposition on the target, together with 20% attenuation and 20% laser energy. Each spot was irradiated at 40 random locations for a total of 1000. The measurement was accumulated over all shots to be representative of the complete sample.

### ^1^H Nuclear Magnetic Resonance (NMR)

All samples were measured by a Bruker AVIII-500 instrument with a 5 mm Prodigy TCI probe with deuterated chloroform as the solvent. The spectra were referenced to the residual signal of the solvent chloroform, and chemical shifts are reported in parts per million.

## Experimental Procedures

All reactions and manipulations were performed in a glovebox filled with argon at an oxygen and moisture content below 0.5 ppm or using standard Schlenk and high-vacuum techniques. THF was dried over the Na/K alloy and benzophenone, degassed, and distilled under high vacuum prior to use. Toluene was dried over a sodium–potassium alloy, degassed, and distilled under high vacuum prior to use. Anhydrous DCM (Sigma-Aldrich) was used as received. Pentane was used as received. Propylene oxide (Sigma-Aldrich) and deuterated propylene oxide (Cambridge Isotope) were degassed, stirred over calcium hydride, distilled, and degassed prior to use. 1-*tert*-Butyl-2,2,4,4,4-pentakis(dimethylamino)-2λ5,4λ5-catenadi(phosphazene) (P_2_-*t*-Bu solution in THF 1.9M) and triethylborane (Et_3_B in THF 1.0 M) were diluted with propylene oxide prior to use. Exo-5-norbornene-2-methanol was synthesized from exo-5-norbornene-2-carboxylic acid (Sigma-Aldrich) using an established procedure and dried via evaporation of toluene at 40 °C.^26^ Grubbs third-generation catalyst (Grubbs-III) was synthesized from the second-generation catalyst (Sigma-Aldrich) using an established procedure.^27^

### Macromonomer Synthesis

#### Inner Part

In a 50 mL vial with screw cap and stir bar, exo-5-norbornene-2-methanol and triethylborane were dissolved in propylene oxide, and the polymerization is started via addition of P_2_-*t*-Bu. The reaction was stirred for 20 h. After completion the catalyst precipitates as colorless flakes. The suspension was diluted with pentane, filtered through basic alumina and a 0.22 μm PTFE syringe filter, and dried under a N_2_ stream. The resulting polymer was twice dried via evaporation of toluene at 40 °C under high vacuum and directly used for the second step.

#### Outer Part

In a 50 mL vial with screw cap and stir bar the already placed inner part of the macromonomer and triethylborane were dissolved in propylene oxide, and the polymerization is started via addition of P_2_-*t*-Bu. The reaction mixture was stirred for 20 h. After completion, the catalyst precipitates as colorless flakes. The suspension was diluted with pentane, filtered through basic alumina and a 0.22 μm PTFE syringe filter, and dried under a N_2_ stream.

### Bottlebrush Synthesis

In a 100 mL flask, the macromonomer was dissolved in DMC as a 0.1 g/mL solution, and Grubbs-III catalyst was added as a 2 mg/mL solution. The reaction is stirred for 15 min, terminated via addition of 1 mL ethyl vinyl ether, diluted with acetone, filtered through basic alumina and a 0.22 μm PTFE syringe filter, and dried under a N_2_ stream. Traces of residual free PPO are removed via three consecutive fractional precipitations by dissolving the polymer in a pentane, with minimal amounts of acetone added to enable solubility at RT, and cooled to −20 °C. The supernatant was discarded, and the precipitated polymer was dried under vacuum at 50 °C for 3 days and prepared for measurement in a glovebox.

## Results and Discussion

Quasi-elastic neutron scattering data are combined among two different instruments and measured for two different temperatures, *T* = 325 K and *T* = 350 K. This combination allows one to capture the segmental dynamics of the side chains in the available time window.

**Figure 2:**
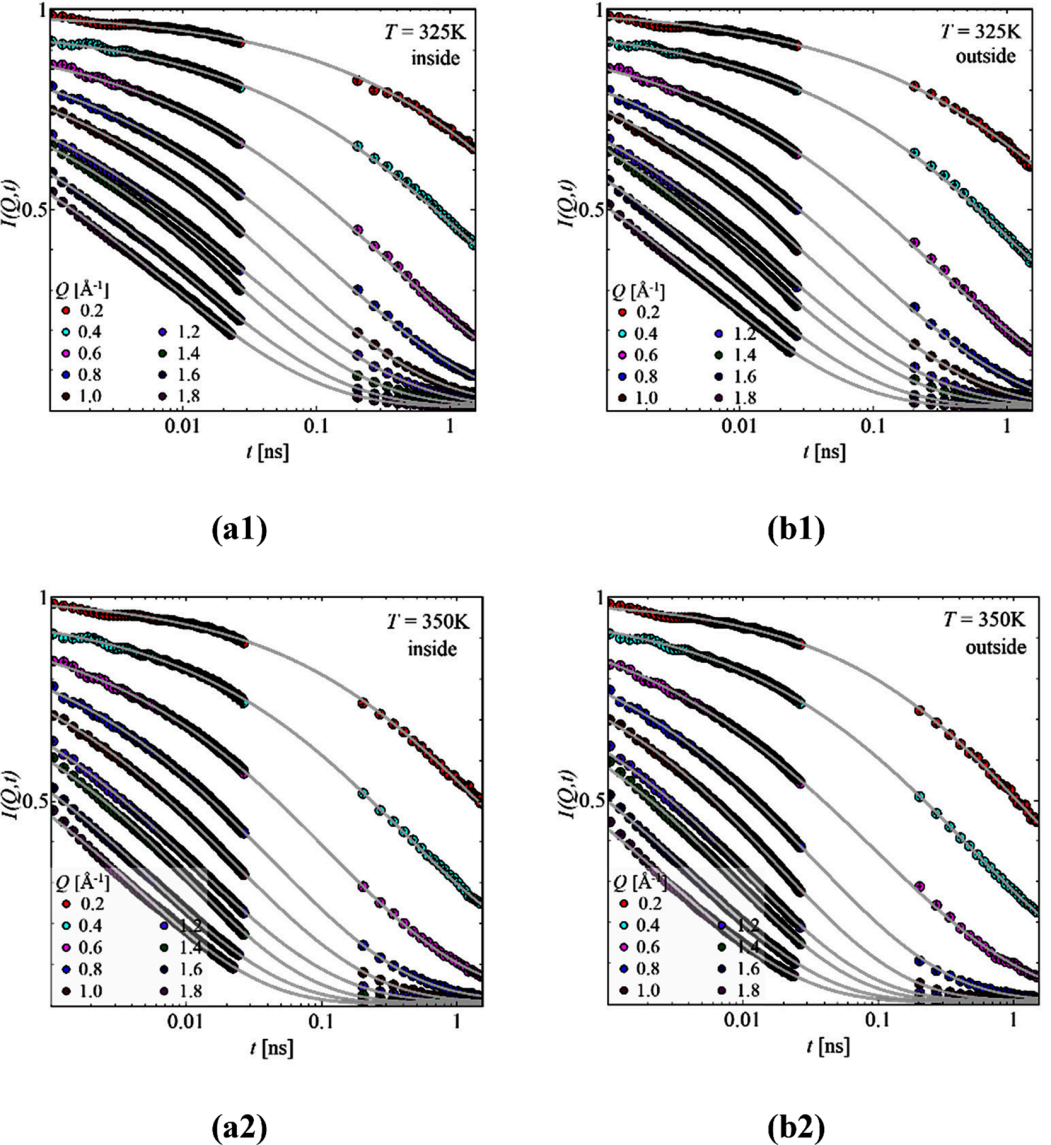
Intermediate scattering function, *I*(*Q,t*), as a function of time, *t*, for *T* = 325 K (top) and *T* = 350 K (bottom) for the samples inside (a1, a2) and outside (b1, b2) for the different *Q* values as indicated. Solid lines are the best description with eq 3.

Figure 2 shows the time-dependent intermediate scattering function, *I*(*Q,t*), for our two samples with labeled parts at (a) inside and (b) outside. Both temperatures have a well pronounced decay, which intensifies for the higher temperature, *T* = 350 K. This indicates that the dynamics progresses through the available time window.

The scattering data (Figure 2) contain information regarding the segmental relaxation of the highlighted parts within the side chains. This can be well described by eq 3. It leads to the *Q*-dependent relaxation time, τ(*Q*), associated with the *Q*-dependent stretching parameter, β(*Q*). For having a better comparison across the different samples and temperatures, eq 4 was employed giving the average relaxation time, .

**Figure 3:**
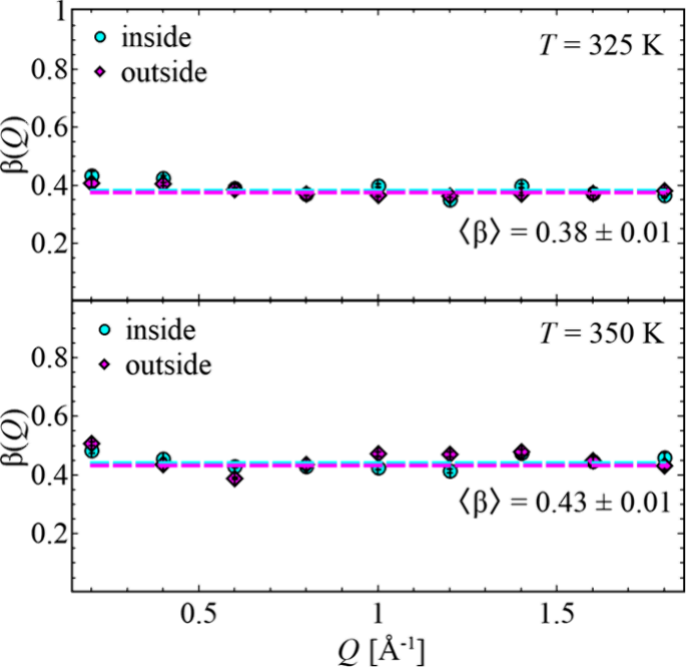
Stretching parameter, , as a function of momentum transfer, *Q*, for *T* = 325 K (top) and *T* = 350 K (bottom) for both samples. Dashed lines are the average values for each sample.

As seen in Figure 3, the stretching parameters for the different samples are on average constant for one temperature across all the *Q* values, but different with respect to *T*. While at *T* = 325 K, , the stretching parameter increases to  at the higher temperature, *T* = 350 K.

In general,  indicates a single-exponential decay, transferring to a Debye-like relaxation behavior with one uniform relaxation time. In polymers, the segmental relaxation itself is of non-Debye nature, which is indicated by .^28^

Setting the obtained values of  in context to a distribution of relaxation times and comparing it with the ideal relaxation process, it is obvious that larger  values, as seen for the higher temperature, indicate a more uniform relaxation.^16^ However, contrary to an assumption of gradual relaxation, for the different locations within the side chains, there is no difference in the distribution of relaxation time, as seen at the on average constant  value.

**Figure 4:**
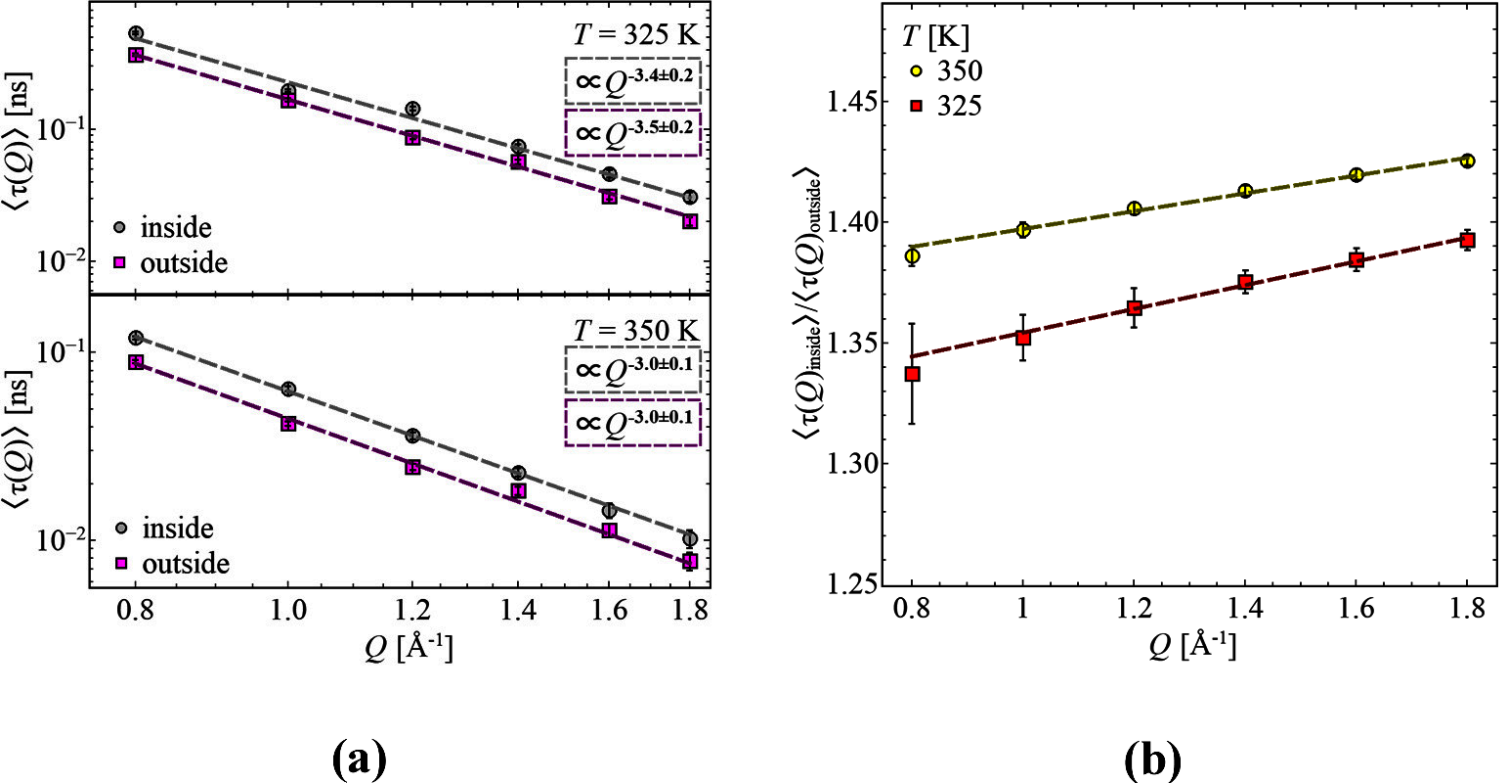
(a) Average relaxation time, , as a function of momentum transfer, *Q*, for the sample inside and outside at temperatures *T* = 325 K (top) and *T* = 350 K (bottom). Dashed lines are the best description with a power law. (b) Ratio of average relaxation times, , as a function of momentum transfer, *Q*, for *T* = 325 K and *T* = 350 K. Dashed lines are the best description with a linear function.

Continuing with the average relaxation times, , of the samples inside and outside, a pronounced *Q* dependence with a power law behavior is seen (Figure 4a). Across both samples, the exponent stays constant within one temperature and slightly increases with increasing temperature. Overall, a reduction in relaxation time is seen while comparing the outside to the inside of the attached side chains. This effect was hypothesized as the grafting process mostly affects segments close to the backbone and is assumed to restrict their relaxation ability. For a better judgment regarding the actual effect, the ratio of  for both temperatures is considered (Figure 4b).

Here it is obvious that at higher temperature the slowdown of the process is more pronounced, resulting in a position-dependent temperature dependence. Also, an increase is visible while increasing the momentum transfer, which indicates a stronger reduction on a local length scale.

As stated earlier, the samples have 10 protonated monomeric units within the side chains, while the remaining 14 or 17 units are deuterated and do not significantly contribute to the scattering signal. Therefore, it is reasonable that the segmental relaxation, based on the power law dependence as illustrated in Figure 3a, can be classified as heterogeneous. This might have the origin in the combination of the very short, protonated part of the side chain giving the scattering signal together with the bottlebrush nature itself. Previous studies on bottlebrush polymers also report heterogeneous dynamics for the segmental relaxation.^4,16,18^ In the case of homogeneous dynamics, the power law for  vs *Q* needs to be −4.6 for *T* = 325 K and −5.3 for *T* = 350 K, based on the relationship of .^29−31^

**Figure 5:**
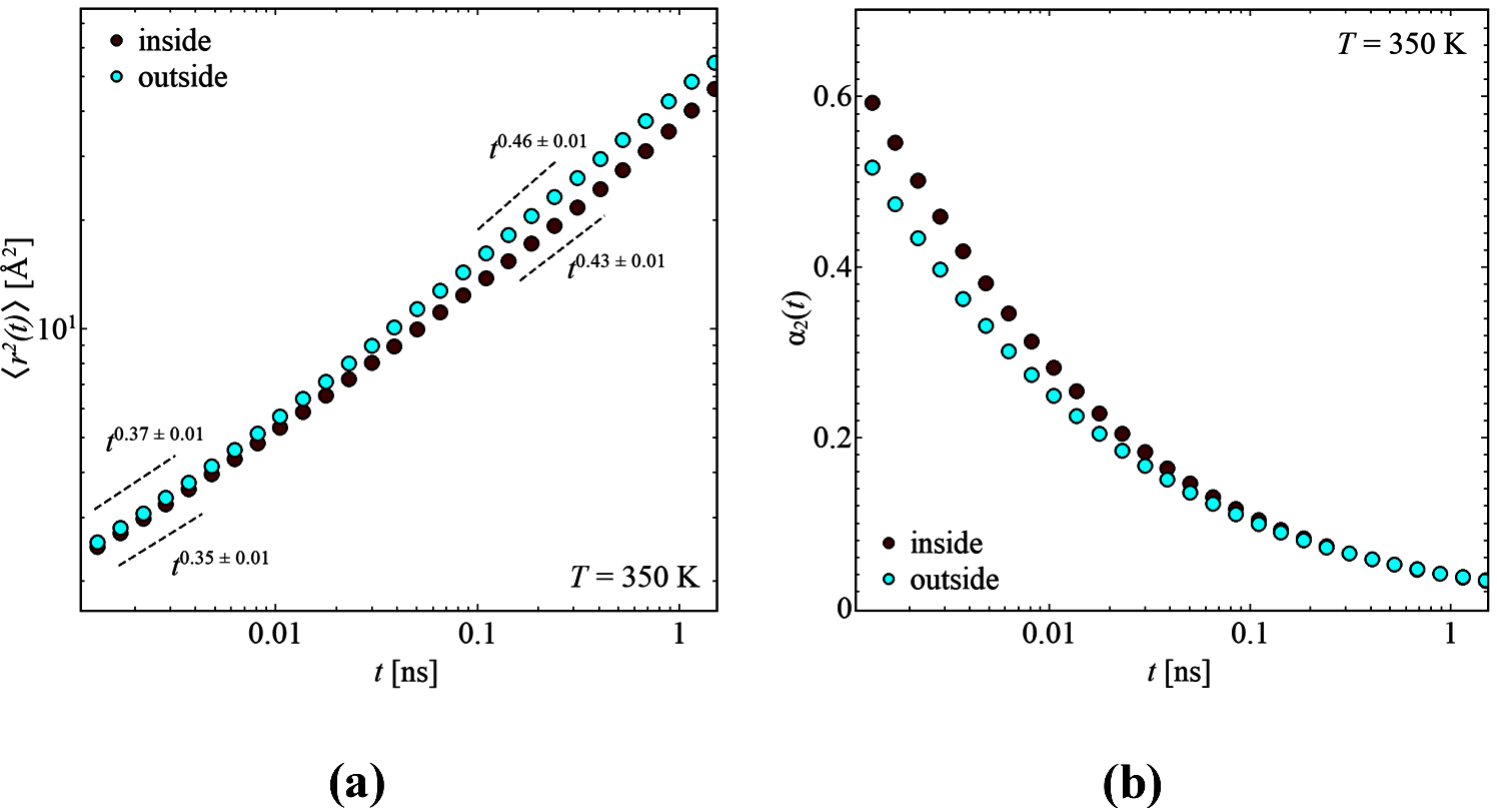
(a) Mean-square displacement, , as a function of time, *t*, and (b) non-Gaussian parameter, , as a function of time, *t*, for the samples labeled on the inside and outside at *T* = 350 K. Dashed lines illustrate the power law proportionality.

Taking the heterogeneous nature of the segmental relaxation into account, the mean-square displacement is extracted considering the non-Gaussian nature, similar to previous work.^4,16^ As seen in Figure 5a, the mean-square displacement increases with time, together with a slight slope change at *t* ∼ 0.01 ns. Comparing both samples shows that the segments close to the backbone are more restricted in their movement, and thus the  is smaller compared to those for the sample having the label on the outside. This is also reflected in the power laws. While at long times the mean-square displacement of the inner segments is proportional to , the segments at the outside propagate with . Especially the power law dependence of the outer segments is very similar to that known for Rouse dynamics of linear polymer, *t*^0.5^, which would indicate a dynamics similar to a free chain.^32^ However, the size of the contributing part of the side chain is very small, for which the Rouse model can usually not be applied, but it can be seen as a rough comparison in regard to the dynamical behavior.

As already noted, the dynamics are of a heterogeneous nature, which is also seen in the non-Gaussian parameter, illustrated in Figure 5b. Here, values of  would indicate Gaussian behavior with homogeneous dynamics. This might be reached at even lower *Q* values, independent of the labeling. For now, the  values are higher for the inner segments, indicating more heterogeneity due to the grafting to the backbone, whereas at longer times the inside and the outside equalize.

**Figure 6:**
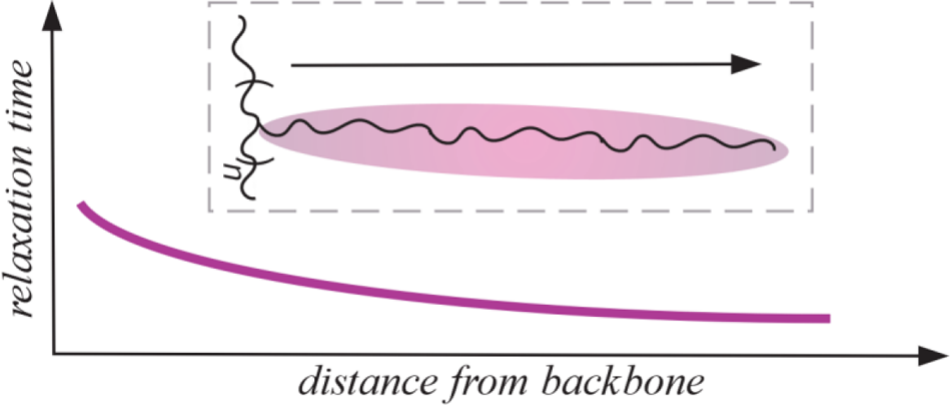
Schematic illustration of relaxation behavior within the side chain. Red parts indicate the inner segments, and black parts indicate the outer part of the side chain.

All results together support our hypothesis that the inner segments are slower and more restricted in their movement compared to the outer segments within the side chain. A simplified illustration of the obtained results regarding the relaxation behavior within the side chain is shown in Figure 6, including the slight gradual relaxation behavior across the entire side chain.

## Summary and Conclusion

We used quasi-elastic neutron scattering and partial deuteration to further follow the segmental dynamics of the side chains from bottlebrush polymers. Previous studies pointed to a gradual relaxation behavior along the segments from the grafting point toward the dangling ends.^4,16^ To elaborate this in more detail, partially protonated samples were used. This means specific parts of the samples were highlighted for quasi-elastic neutron scattering, whereas the contributions from the other parts were minimized. Specifically, each sample has 10 monomeric units protonated, while the remaining 14 or 17 segments are deuterated. The difference across the samples is the location of the protonated parts. It is on either the inside or the outside of the side chain. This allows one to get information about the relaxation behavior from the different parts of the side chain.

Combining all results gives faster dynamics, associated with larger mean-square displacement and less heterogeneity for the outermost segments. Going inward along the side chains toward the backbone, the relaxation slows down and is more restricted due to the tethering to the backbone. This is reflected in the relaxation times as well as in the mean-square displacement by showing lower values. Across both measurement temperatures, this effect is similar to a slight intensifying at the higher temperature, *T* = 350 K.

## Supporting Information Available

GPC and ^1^H results of the precursor and samples (PDF)
