# Supplementary material for: Position-Dependent Segmental Relaxation in Bottlebrush Polymers
Source: Macromolecules. 2024 May 11;57(10):4729–36. doi: 10.1021/acs.macromol.4c00243 (PMC11140752; doi:10.1021/acs.macromol.4c00243)
Supplement: Supplementary file 1 — ma4c00243_si_001.pdf [file ma4c00243_si_001.pdf]

Supporting Information

Position Dependent Segmental Relaxation in Bottlebrush Polymers

Karin J. Bichler[1*], Bruno Jakobi[1], Alice Klapproth[2], Richard A. Mole[2], Gerald J. Schneider[1,3*]

[1] Department of Chemistry, Louisiana State University, Baton Rouge, Louisiana 70803, United States
[2] Australian Nuclear Science and Technology Organisation, New Illawarra Road, Lucas Heights, 2234, NSW, Australia
[3] Department of Physics & Astronomy, Louisiana State University, Baton Rouge, Louisiana 70803, United States

Correspondence: kbichler@lsu.edu, gjschneider@lsu.edu

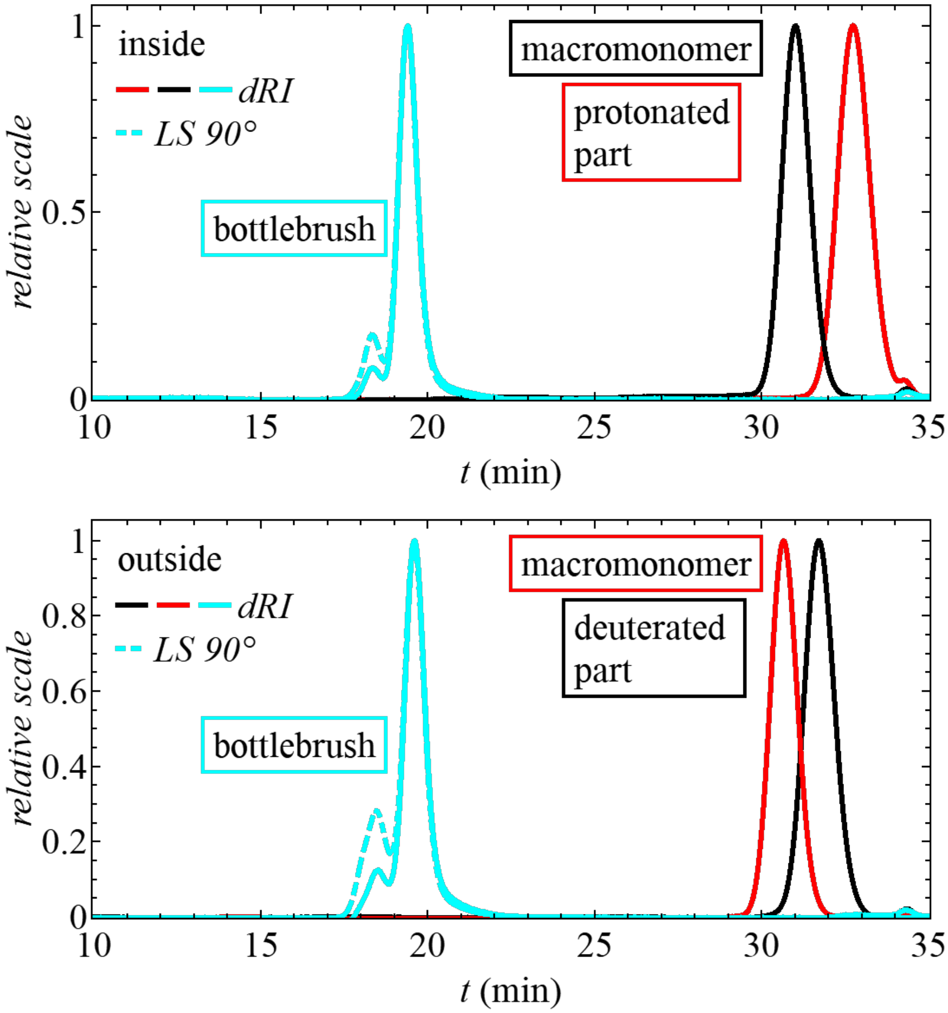

Figure S1: Differential refractive index (dRI) (solid line) and 90° light scattering (90° LS) (dotted line) signal of the GPC chromatogram of all inner parts, macromonomers, and bottlebrushes for the samples inside **(top)** and outside **(bottom)**.

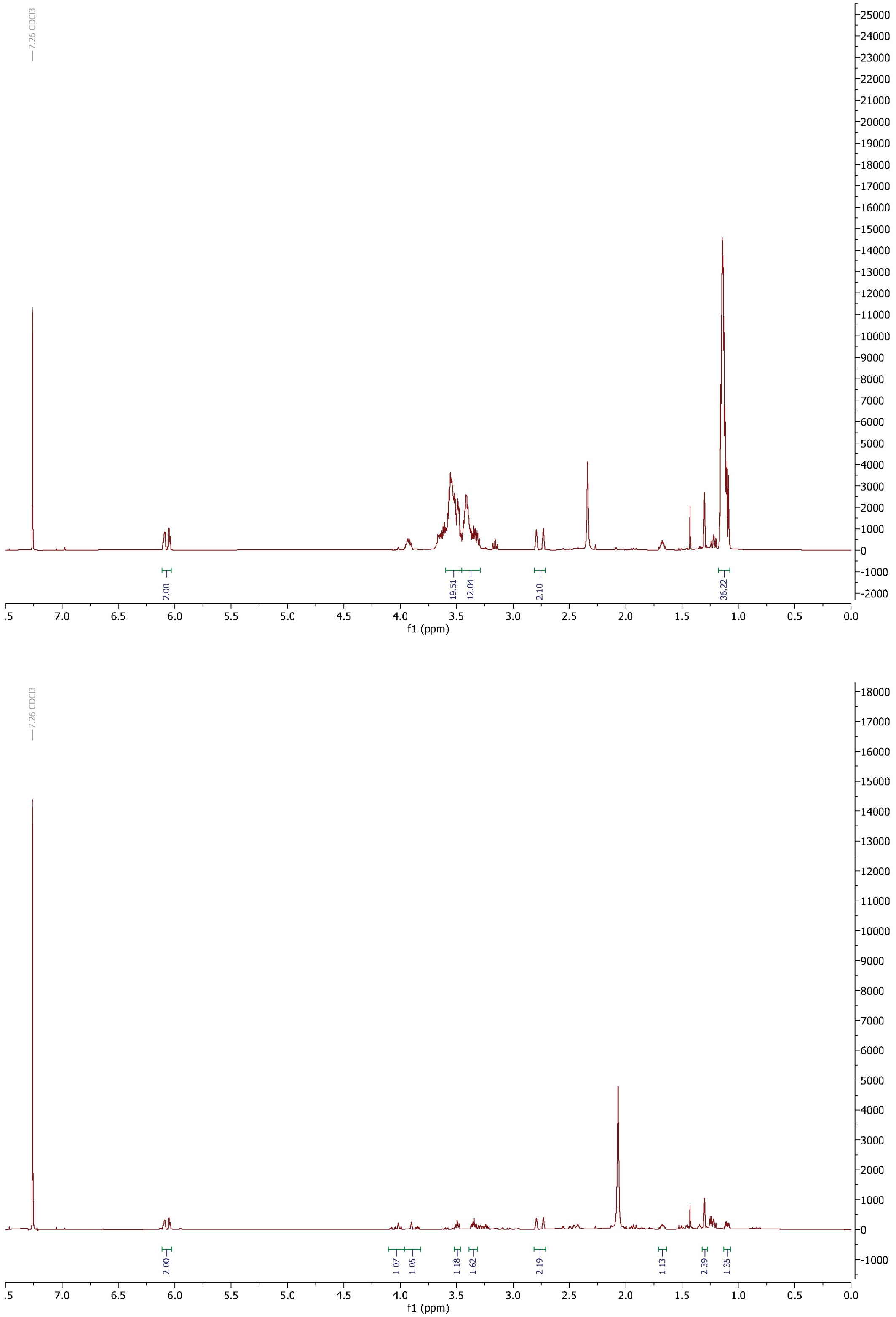

Figure S2: 1H NMR of the inner part of the macromonomer for the sample inside **(top)**, and outside **(bottom)**.

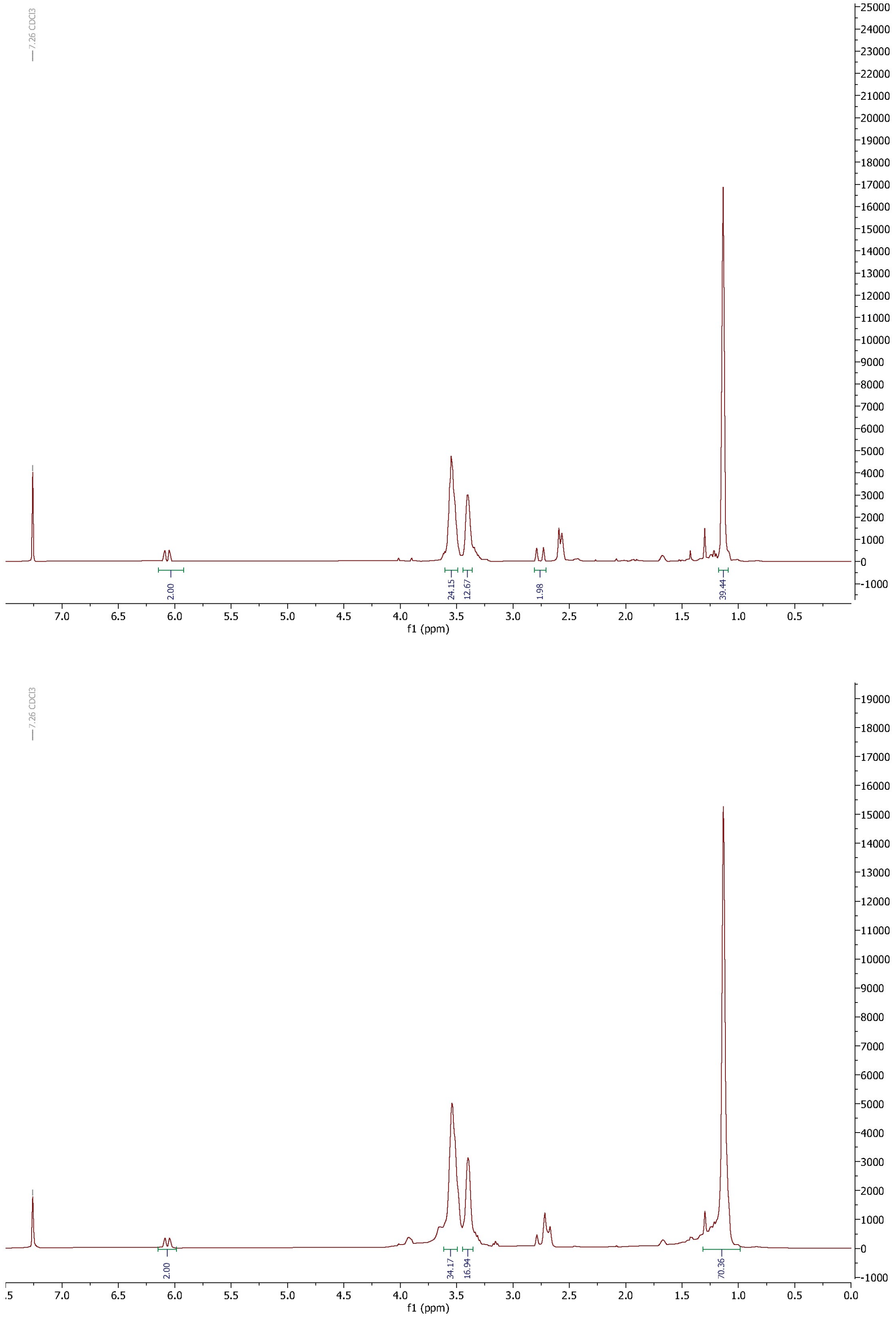

Figure S3: 1H NMR of the complete macromonomer for the sample inside **(top)**, and outside **(bottom)**.

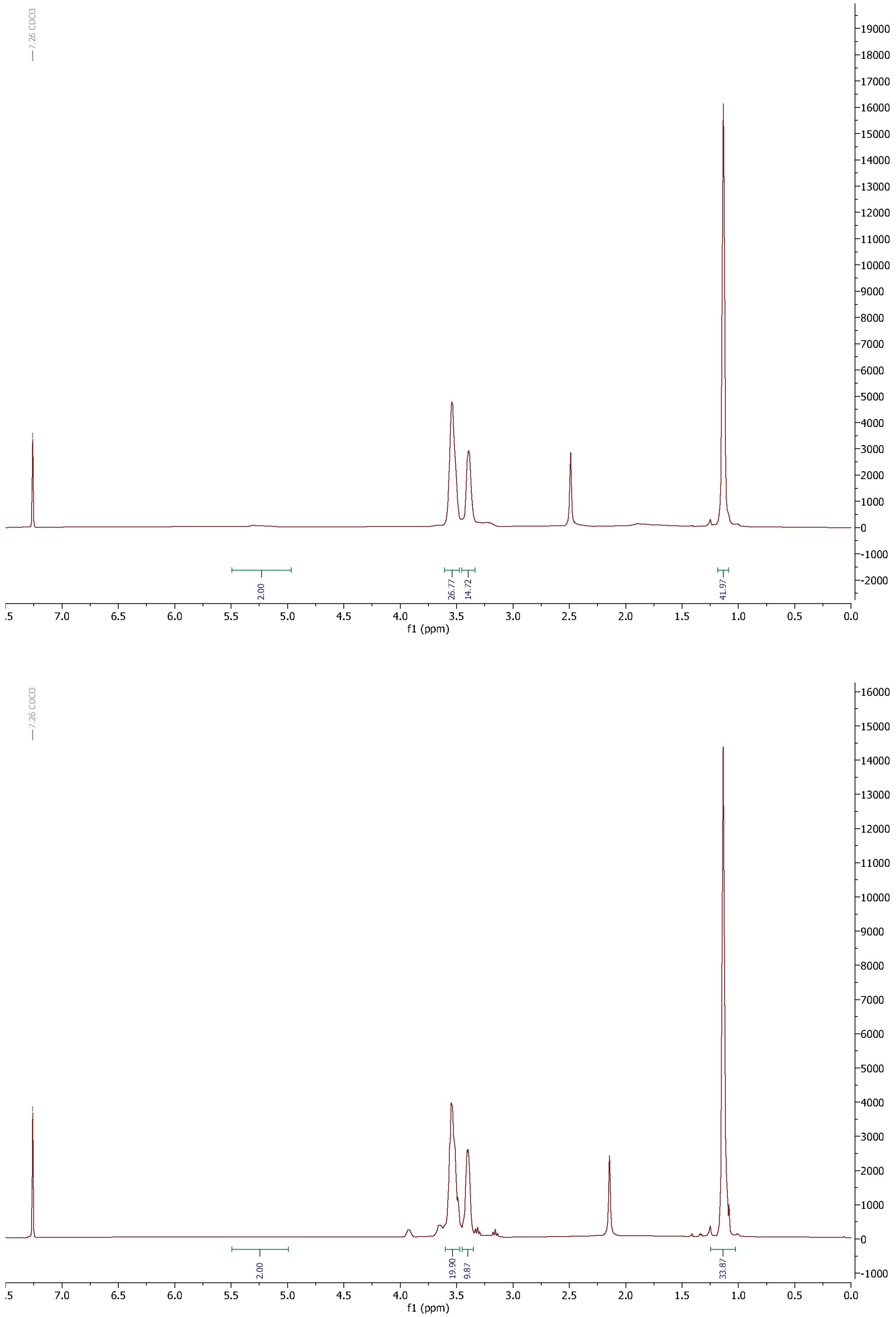

Figure S4: 1H NMR of finished bottlebrush for the sample inside **(top)**, and outside **(bottom)**.
